# Association between oxidative balance score and female infertility from the national health and nutrition examination survey 2013–2018

**DOI:** 10.3389/fendo.2024.1386021

**Published:** 2024-07-30

**Authors:** Zhe Su, Peihui Ding, Wenjing Su, Xia Li, Yiqian Li, Xiaoran Li, Kaixue Lao, Yanlin Wang

**Affiliations:** ^1^ Department of Reproductive Medicine, Binzhou Medical University Hospital, Binzhou, China; ^2^ Department of Radiology, Binzhou Medical University Hospital, Binzhou, China; ^3^ Community Health Service Center of Dudian Street in Bincheng District, Binzhou, China

**Keywords:** oxidative balance score (OBS), female infertility, oxidative stress, NHANES, antioxidants, pro-oxidants

## Abstract

**Background:**

The correlation between oxidative stress and female infertility pathogenesis was established, and the oxidative balance score (OBS) can serve as a measure of overall oxidative stress burden within an individual. Prior reports have not addressed the relationship between OBS and female infertility. This study endeavors to investigate the association between infertility risk in female and OBS.

**Methods:**

The analysis focused on data from the National Health and Nutrition Examination Survey 2013-2018. OBS was determined from 16 dietary components and 4 lifestyle components. Multivariate logistic regression was employed to investigate the relationship between OBS and female infertility. Further stratified analysis was conducted to examine the associations across various subgroups. To elucidate the dose-response relationship between infertility risk in female and OBS, a restricted cubic spline function was employed.

**Results:**

The study included a total of 1410 participants. Through weighted multivariable logistic regression analysis, we observed a consistent inverse correlation between OBS and the risk of female infertility [OR (95% CI) = 0.97 (0.95, 0.99), p = 0.047]. When participants were segregated into quartiles based on OBS, those in the highest quartile had a 61% [OR (95% CI) = 0.39 (0.2, 0.79), p = 0.01] reduced risk of infertility compared to those in the lowest quartile of OBS. A trend test assessing OBS by quartile also revealed the relationship between OBS and female infertility. This correlation remained constant across both dietary and lifestyle OBS. Additionally, lifestyle OBS and female infertility exhibited a nonlinear association. A sensitivity analysis verified the consistency of our findings.

**Conclusion:**

The study found that a higher OBS is associated with a lower prevalence of female infertility. These results emphasized the potential role of oxidative homeostasis in the pathogenesis of infertility and highlighted the importance of follow-up studies and prevention strategies.

## Introduction

Infertility is characterized clinically as a reproductive system disorder, the inability to realize a clinical pregnancy following a year of consistent, unprotected sexual activity (1). The prevalence of infertility is increasing, and the latest World Health Organization study on sexual and reproductive health shows that the global prevalence of infertility in 2022 is estimated to be about one in six people worldwide experiencing infertility at some point in their lives, based on data from 1990 to 2021. Specifically, the lifetime prevalence of infertility is currently estimated at 17.5 percent (2). A study conducted in Europe has demonstrated the significant economic burden of infertility, with an annual expenditure of 70 million euros per 10000 women aged 18 to 50 years old (3). Therefore, infertility has emerged as a substantial medical and societal issue, with a significant effect on global health and placing a substantial burden on both individuals and society (4). The World Health Organization has categorized infertility as a societal disorder, and the U.S. Centers for Disease Control and Prevention (CDC) has designated infertility as a public health priority (5, 6).

Infertility has a multitude of causes, and factors affecting female fertility may comprise tubal disease, anovulation, endometriosis, pelvic adhesions, and unexplained infertility (7). Among these, poor oocyte quality stands out as a primary cause of female infertility (8). Oxidative stress (OS) plays a critical role in oocyte aging, and the accumulation of reactive oxygen species (ROS) during reproductive aging is implicated in oocyte damage and infertility (9). ROS-induced OS is identified as a primary factor in causing female subfertility (10). Although ROS are continually produced in the mitochondria of aerobic organisms, antioxidant enzymes are also active in eliminating them, thereby preserving redox equilibrium and homeostasis. Nevertheless, an imbalance in the generation of ROS and the capacity of antioxidants can cause the accumulation of ROS, subsequently leading to a range of reproductive illnesses, including polycystic ovary syndrome (PCOS), endometriosis, and unexplained infertility (10, 11).

OS is defined as a disproportion between antioxidant defense systems and pro-oxidant molecules (12). The dietary intake of individuals serves as a crucial source of both antioxidants and pro-oxidants. The connection between OS and infertility has become a topic of interest for researchers. In recent years, a plethora of studies have examined the correlation between various antioxidants and the prevalence of infertility (13–15). Alongside dietary factors, several lifestyle aspects such as smoking, alcohol consumption, physical activity, and obesity also influence OS and fertility (16–20).

To provide a holistic assessment of an individual’s exposure to both pro-oxidants and antioxidants, the Oxidative Balance Score (OBS) is utilized. It describes the degree of exposure related to OS based on the sum of the various pro-oxidants and antioxidants intakes by assigning corresponding scores to each of its components and summing them. The original OBS was created by Van Hoydonck et al., which included just three components, two antioxidants (β-carotene and vitamin C) and one pro-oxidant (iron) (21). Subsequently, OBS components were enriched and broadened. Over 20 variations of these OBSs have now been released, in an effort to refine this assessment by selecting diverse components or by adopting different scoring systems (22). This comprehensive metric primarily takes into account dietary and lifestyle components. A higher OBS indicates greater antioxidant and reduced pro-oxidant exposure, implying a lower level of OS. Prior research has affirmed that elevated OBS can decrease the risk of certain diseases. For instance, they have been linked to a lower prevalence of nonalcoholic fatty liver disease (23), as well as a reduction in stroke prevalence (24), and enhancement in cognitive function (25), among others. Nevertheless, the association between OBS and infertility risk in female has not been explored. Hence, leveraging the National Health and Nutrition Examination Survey (NHANES) 2013-2018 data, we conducted a cross-sectional study on the relationship between OBS and female infertility. This investigation could enhance the comprehension of the impact of OS on female infertility development and potentially put forward some innovative ideas for new preventive strategies.

## Methods

### Study population

The NHANES is a sequence of cross-sectional surveys of U.S. citizens featuring multi-stage, complex probability sampling that are nationally representative. It has been subjected to ethical review and approval by the National Center for Health Statistics Ethics Review Committee, and every participant involved in the survey provided informed consent. Additional information is available on the official website.

**Figure 1:**
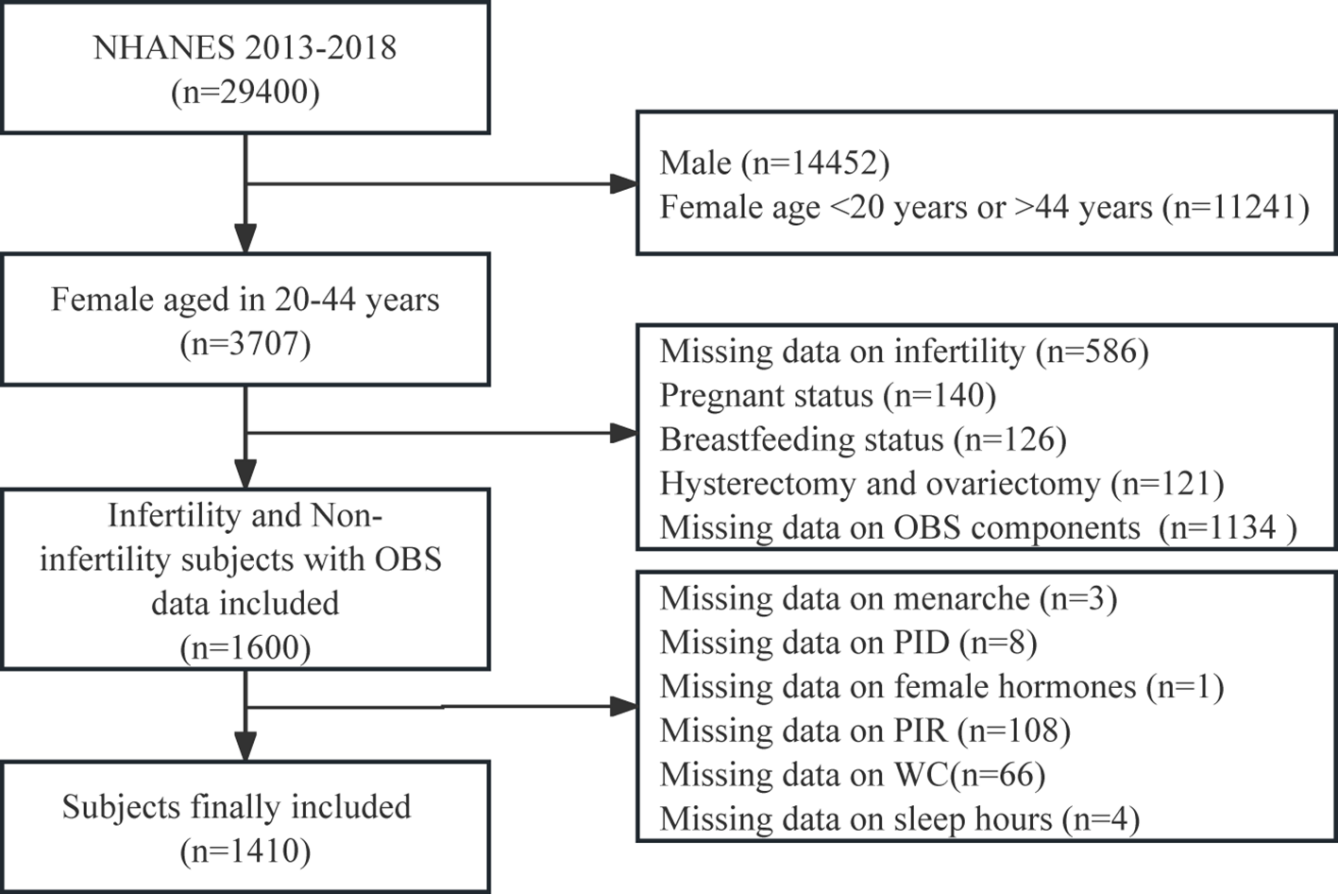
Flowchart of the study population. NHANES, National Health and Nutrition Examination Survey; OBS, oxidative balance score.

NHANES administers a nationwide survey of U.S. adults and children every two years, however, only three complete 2-year survey cycles (2013–2014, 2015–2016, and 2017–2018) contain relevant information on female infertility. For our research, we analyzed data from three consecutive cycles of the NHANES survey spanning from 2013 to 2018, initially encompassing 29400 participants. We implemented various exclusion criteria: we initially omitted males (n = 14452) and females younger than 20 years old or older than 44 years old (n = 11241). Subsequently, we excluded any individuals with missing data on infertility (n = 586) and OBS components (n = 1134). Additionally, participants who were pregnant (n = 140) or breastfeeding (n = 126), as well as those with other conditions such as hysterectomy and ovariectomy (n = 121), were also excluded. Finally, we excluded populations with other potential causes of infertility or missing data on covariates, which included age at menarche (n = 3), treatment for pelvic inflammatory disease (PID) (n = 8), female hormones use (n = 1), family income to poverty ratio (PIR) (n = 108), waist circumference (WC) (n = 66), and sleep hours on workdays (n = 4). The ultimate tally of participants amounted to 1410 individuals (**Figure 1**).

### Exposure and outcome definitions

The outcome variable was whether the participants had infertility. NHANES has included information related to infertility in its questionnaire since 2013. The assessment of infertility adhered to the methodologies detailed in existing publications (26–28). Participants were assessed for self-reported infertility by providing an affirmative response to these questions such as “Have you ever attempted to become pregnant over a period of at least a year without becoming pregnant?” or “Have you ever been to a doctor or other medical provider because you have been unable to become pregnant?”. Those who responded “yes” to these inquiries were grouped as possessing a record of infertility.

Based on the available data and its relation to OS, we utilized the most recent scoring method from prior studies to assess the OBS (23, 29), consisting of 16 dietary and 4 lifestyle components. Among these components, we identified pro-oxidants, which included total fat, cotinine, alcohol consumption, and body mass index. On the other hand, antioxidants including dietary fiber, β-carotene, vitamin B_2_, niacin, vitamin B_6_, total folate, vitamin B_12_, vitamin C, vitamin E, calcium, magnesium, zinc, copper, selenium, iron, and physical activity were identified. To estimate dietary intake, we conducted two 24-hour dietary surveys and calculated the average intake based on the mean of these two interviews. The lifestyle components encompassed cotinine levels, alcohol consumption, body mass index (BMI), and physical activity (PA). Serum cotinine, a nicotine by-product, served as an indicator for assessing exposure to tobacco smoke, covering both active smoking and passive smoking. Alcohol consumption data was collected via the question “During the past 12 months, on those days that you drank alcoholic beverages, on the average, how many drinks did you have?”. All participants had their body measurements taken by trained examiners. PA was calculated by multiplying the weekly frequency of each activity by its duration and further multiplying by the corresponding metabolic equivalent score, encompassing work-related activity, walking or bicycling for transportation, and leisure-time PA. For work-related activity or leisure-time PA, minutes of vigorous PA were doubled and added to minutes of moderate PA, and then multiplied by the number of days of activity to calculate the total minutes of PA spent in a typical week (30, 31). All components were distributed into three groups based on weighted tertiles. Antioxidant components were assigned scores of 2, 1, and 0 from the highest to lowest tertiles, while pro-oxidant components were scored conversely, from 0 to 2, across the same tertiles. The total OBS, which is the cumulative score of all these components, reflects a higher exposure to antioxidants as the score increases. **Table 1** presented the categorization and assigned scores for each OBS component.

**Table 1:** Oxidative Balance Score assignment scheme.

OBS components	Property	Scoring assignment		
Dietary components		0	1	2
Dietary fiber (g/d)	Antioxidant	<11.15	11.15-17.45	>17.45
β-carotene (RE/d)	Antioxidant	<587.5	587.5-2115.5	>2115.5
Riboflavin (Vitamin B_2_) (mg/d)	Antioxidant	<1.406	1.406-2.1065	>2.1065
Niacin (mg/d)	Antioxidant	<17.7935	17.7935-25.196	>25.196
Vitamin B_6_ (mg/d)	Antioxidant	<1.386	1.386-2.015	>2.015
Total folate (mcg/d)	Antioxidant	<260.5	260.5-377.5	>377.5
Vitamin B_12_ (mcg/d)	Antioxidant	<2.525	2.525-4.275	>4.275
Vitamin C (mg/d)	Antioxidant	<31.8	31.8-86.15	>86.15
Vitamin E (ATE) (mg/d)	Antioxidant	<5.97	5.97-9.35	>9.35
Calcium (mg/d)	Antioxidant	<660	660-982	>982
Magnesium (mg/d)	Antioxidant	<220	220-297	>297
Zinc (mg/d)	Antioxidant	<7.35	7.35-10.35	>10.35
Copper (mg/d)	Antioxidant	<0.8505	0.8505-1.208	>1.208
Selenium (mcg/d)	Antioxidant	<79.4	79.4-113.35	>113.35
Total fat (g/d)	Pro-oxidant	>82.73	58.235-82.73	<58.235
Iron (mg/d)	Antioxidant	<9.29	9.29-13.305	>13.305
Lifestyle components		0	1	2
Cotinine (ng/mL)	Pro-oxidant	>0.171	0.016-0.171	<0.016
Alcohol (g/d)	Pro-oxidant	>3	2-3	<2
Body mass index (kg/m^2^)	Pro-oxidant	>31	23.5-31	<23.5
Physical activity (MET-minute/week)	Antioxidant	<1680	1680-4800	>4800

OBS, Oxidative Balance Score; RE, retinol equivalent; ATE, alpha-tocopherol equivalent; MET, metabolic equivalent.

### Covariates

The covariates selection was informed by prior research on risk factors for reproductive health (32–35). This study included age, total energy intake, BMI, WC, sleep hours on workdays, age at menarche, ethnic background, marital status, education, PIR, drinks (yes, no), smoking (yes, no), total PA (yes, no), regular periods (yes, no), PID (yes, no), trouble sleeping (yes, no), female hormones use (yes, no), birth control pills use (yes, no), and reproductive history (yes, no, and missing) as covariates. The reproductive history was defined as having given birth, either vaginally or by caesarean section, and in addition, both stillbirths and live births were counted. Alcohol consumption status was determined as having a minimum of 12 drinks annually (36). Smoking status was determined using the serum cotinine level, with participants having a cotinine level of 3 ng/ml or above being classified as smokers (37). Based on the 2018 Physical Activity Guidelines Advisory Committee Scientific Report, total minutes of PA were further categorized as 150 minutes or more or 0-149 minutes, representing meeting versus not meeting PA guidelines, respectively (38). Additionally, other covariates were extracted from the NHANES database, including demographic information, examination results, and questionnaire responses.

### Statistical analysis

Following the guidance of the CDC, all statistical analyses were conducted by taking into account the suitable NHANES sampling weights and the complexity of multistage cluster surveys. During the descriptive analysis, the two groups classified based on infertility status were compared using either a weighted Wilcoxon rank-sum test (for continuous variables) or a weighted Rao-Scott chi-square test (for categorical variables) to examine differences in the distribution of sociodemographic and lifestyle behavioral characteristics. The weighted median values, along with interquartile ranges, were reported to describe continuous variables. For categorical variables, frequency and weighted percentage were presented. The inclusion of weighting variables in the statistical analysis served to enhance the representativeness of the population. Variables with a higher incidence of missing values such as reproductive history (n = 494) were designated as “missing”.

To explore the correlation between OBS and female infertility, we carried out the analysis of survey-weighted multivariable logistic regression and calculated the odds ratio (OR) values along with 95% confidence intervals (95% CI). Three multivariate test models were developed. Model 1 had no adjusted variables. Model 2 accounted for age, PIR, smoking, and drinks. Model 3 further adjusted for PID and female hormones factors, based on Model 2. Separate analyses were performed to appraise the relationships between dietary OBS, lifestyle OBS, and infertility risk in female. To gauge its robustness, we transformed the continuous variable OBS into a categorical variable divided into quartiles for subsequent analysis. The Restricted Cubic Spline (RCS) method with four knots placed at the 5^th^, 35^th^, 65^th^, and 95^th^ percentiles was employed to examine any nonlinear associations between OBS and female infertility prevalence based on Model 3. Trend tests were utilized treating the OBS categories as continuous variables to investigate the linear trend association between OBS and female infertility. Subgroup analysis was performed to investigate the OBS and infertility relationship in different age groups, PIR, smoking, drinks, PID, and female hormones variables, with interaction tests examining the consistency of associations across subgroups. Finally, a sensitivity analysis was conducted through stepwise exclusion of each OBS component to further evaluate the robustness of our findings.

The statistical significance level used in this study was determined as two-sided with a P-value less than 0.05. All statistical analyses were performed using R software (version 4.2.1) and appropriate packages.

## Results

### Baseline characteristics

A comparison of the basic characteristics of participants with and without fertility issues was presented in **Table 2**. The present study included a total of 1410 participants from NHANES, representing around 29 million noninstitutionalized U.S. residents, with the majority being non-Hispanic white. Among the participants, 184 (14%) were diagnosed with infertility. All parameters, including age, BMI, WC, lifestyle OBS, marital status, PID, trouble sleeping, female hormones use, previous reproductive history, OBS quartile, and lifestyle OBS quartile were significantly different (all P < 0.05). Females experiencing infertility were older (35 vs 30, P < 0.001) with higher BMI (31 vs 26, P < 0.001) and WC (100 vs 88, P < 0.001) compared to those without infertility. Additionally, they were more intend to suffer from PID (10% vs 3.1%, P = 0.005), more inclined to have trouble sleeping (39% vs 23%, P = 0.008), and more ever use female hormones (9.8% vs 2.7%, P = 0.017) compared to those without infertility. On the other hand, the patients with infertility were more likely to be married (54% vs 38%, P < 0.001) and had a reproductive history (69% vs 53%, P = 0.007). Furthermore, infertility patients had higher family income levels (PIR >3.5, 42% vs 39%, P = 0.4), fewer hours of sleep on workdays (7.50 vs 7.59, P = 0.2), and a higher proportion of smoking (32% vs 24%, P = 0.1) and drinks (2.8% vs 0.8%, P = 0.091) compared to non-infertility patients, and although not statistically significant, there were observable differences. Lastly, females with infertility demonstrated lower OBS (20 vs 21, P = 0.064), dietary OBS (15 vs 16, P = 0.2), and lifestyle OBS (3 vs 4, P = 0.008). **Supplementary Table S1** detailed the demographic and clinical characteristics of the participants by OBS quartile. Comparing the top and bottom OBS quartile, individuals in the latter were more likely to be infertile (8.5% vs 19%, P = 0.011). Regarding socioeconomic status, those with higher OBS tended to exhibit higher levels of educational attainment (college graduate or above, 51% vs 19%, P < 0.001) and income (PIR >3.5, 50% vs 33%, P = 0.009), and a lower proportion of these individuals were smokers (12% vs 39%, P < 0.001) and drinkers (0.1% vs 0.5%, P = 0.071) in terms of lifestyle. Additionally, participants with higher OBS experienced fewer sleep issues (19% vs 34%, P = 0.004), higher energy intake (2291 vs 1338, P < 0.001), and lower BMI (25 vs 29, P < 0.001) and WC (85 vs 98, P < 0.001).

**Table 2:** Basic characteristics of participants by infertility and non-infertility.

Characteristic	Total, N = 1410(100%)^1^	Infertility, N = 184 (14%)^1^	Non-infertility, N = 1226 (86%)^1^	P-value^2^
**Weighted number**	29231894	4096862	25135032	
**Age (years)**	31 (25,37)	35 (29,40)	30 (25,36)	**<0.001**
**Energy (kcal)**	1771 (1410,2205)	1842 (1410,2187)	1767 (1408,2207)	>0.9
**BMI (kg/m^2^)**	27 (22,33)	31 (25,37)	26 (22,33)	**<0.001**
**WC (cm)**	90 (80,104)	100 (89,117)	88 (79,103)	**<0.001**
**Sleep hours on workdays**	7.50 (7,8.5)	7.50 (6,8)	7.59 (7,8.5)	0.2
**Menarche (years)**	13 (12,13)	12 (11,13)	13 (12,13)	0.3
**OBS**	21 (13, 27)	20 (12, 25)	21 (13, 27)	0.064
**OBS Dietary**	16 (10, 22)	15 (9, 21)	16 (10, 23)	0.2
**OBS Lifestyle**	4 (3, 5)	3 (2, 5)	4 (3, 5)	**0.008**
**Race/ethnicity**				0.9
*Mexican American*	216 (11%)	31 (13%)	185 (11%)	
*Non-Hispanic Asian*	147 (5.2%)	17 (3.5%)	130 (5.5%)	
*Non-Hispanic Black*	291 (12%)	43 (12%)	248 (12%)	
*Non-Hispanic White*	553 (61%)	72 (60%)	481 (62%)	
*Other Hispanic*	120 (6.1%)	10 (6.0%)	110 (6.1%)	
*Other Race*	83 (4.8%)	11 (5.8%)	72 (4.7%)	
**Marital status**				**<0.001**
*Living with partner*	203 (13%)	20 (7.8%)	183 (14%)	
*Married*	527 (40%)	101 (54%)	426 (38%)	
*Never married*	514 (36%)	39 (21%)	475 (38%)	
*Separated*	55 (2.9%)	5 (2.3%)	50 (3.0%)	
*Widowed*	10 (1.1%)	3 (6.1%)	7 (0.3%)	
*Divorced*	101 (6.8%)	16 (8.8%)	85 (6.5%)	
**Education**				0.4
*Less than 9th grade*	32 (1.3%)	2 (0.7%)	30 (1.5%)	
*9-11th grade*	105 (5.2%)	12 (5.2%)	93 (5.2%)	
*High school graduate/GED*	247 (16%)	41 (20%)	206 (16%)	
*Some college or AA degree*	577 (39%)	82 (44%)	495 (38%)	
*College graduate or above*	449 (38%)	47 (31%)	402 (39%)	
**PIR**				0.4
*<1.3*	433 (25%)	52 (20%)	381 (26%)	
*1.3-3.5*	529 (35%)	65 (38%)	464 (35%)	
*>3.5*	448 (40%)	67 (42%)	381 (39%)	
**drinks**				0.091
*No*	1398 (99%)	180 (97%)	1218 (99%)	
*Yes*	12 (1.1%)	4 (2.8%)	8 (0.8%)	
**smoking**				0.1
*No*	1036(75%)	122 (68%)	914 (76%)	
*Yes*	374 (25%)	62 (32%)	312 (24%)	
**Total PA**				0.15
*No*	193 (11%)	26 (15%)	167 (10%)	
*Yes*	1217 (89%)	158 (85%)	1059 (90%)	
**regular periods**				0.8
*No*	77 (5.2%)	11 (4.8%)	66 (5.3%)	
*Yes*	1333 (95%)	173 (95%)	1160 (95%)	
**PID**				**0.005**
*No*	1345 (96%)	169 (90%)	1176 (97%)	
*Yes*	65 (4.1%)	15 (10%)	50 (3.1%)	
**trouble sleeping**				**0.008**
*No*	1065 (75%)	118 (61%)	945 (77%)	
*Yes*	345 (25%)	66 (39%)	279 (23%)	
**female hormones**				**0.017**
*No*	1372 (96%)	174 (90%)	1198 (97%)	
*Yes*	38 (3.7%)	10 (9.8%)	28 (2.7%)	
**birth control pills**				0.4
*No*	374 (21%)	38 (18%)	336 (22%)	
*Yes*	1036 (79%)	146 (82%)	890 (78%)	
**reproductive history**				**0.007**
*No*	76 (5.6%)	19 (9.3%)	57 (5.0%)	
*Yes*	840 (55%)	129 (69%)	711 (53%)	
*Missing*	494 (39%)	36 (22%)	458 (42%)	
**OBS Quartile**				**0.011**
*Q1 (<13)*	343 (23%)	45 (31%)	298 (22%)	
*Q2 (13-20)*	376 (26%)	53 (22%)	323 (27%)	
*Q3 (21-26)*	350 (25%)	53 (32%)	297 (24%)	
*Q4 (≥27)*	341 (26%)	33 (15%)	308 (27%)	
**OBS Dietary Quartile**				0.4
*Q1 (<10)*	374 (25%)	49 (30%)	325 (24%)	
*Q2 (10-15)*	297 (22%)	44 (20%)	253 (22%)	
*Q3 (16-21)*	338 (24%)	45 (26%)	293 (24%)	
*Q4 (≥22)*	401 (29%)	46 (25%)	355 (30%)	
**OBS Lifestyle Quartile**				**0.002**
*Q1 (<3)*	251 (16%)	45 (25%)	206 (15%)	
*Q2 (3)*	293 (18%)	42 (27%)	251 (16%)	
*Q3 (4)*	350 (25%)	41 (18%)	309 (27%)	
*Q4 (≥5)*	516 (41%)	56 (30%)	460 (42%)	

^1^median (IQR) for continuous; n (%) for categorical.

^2^Wilcoxon rank-sum test for complex survey samples; chi-squared test with Rao & Scott’s second-order correction.

BMI, body mass index; WC, waist circumference; PIR, ratio of family income to poverty; PA, physical activity; PID, pelvic inflammatory disease; Q, quartile. Values in bold indicate statistical significance.

### Association between oxidative balance score and female infertility

The examination of the association between OBS and female infertility was undertaken by utilizing weighted logistic regression analysis in three distinct models. Subsequently, the results from these models were summarized and displayed in **Table 3**. The findings revealed that higher OBS (continuous) exhibits a negative association with infertility in both Model 2 [OR (95% CI) = 0.97 (0.94, 0.99), p = 0.045] and Model 3 [OR (95% CI) = 0.97 (0.95, 0.99), p = 0.047]. Notably, in Model 3, each one-unit rise in the OBS corresponded to a 3% reduction in the risk of infertility. Additionally, OBS quartile (including lifestyle OBS quartile) was consistently linked to a decreased risk of infertility across all three models, with statistical significance (P < 0.05). When OBS (including lifestyle OBS) was treated as a categorical variable, the overall trend indicated a reduction in the risk of infertility with increasing quartiles in all models (P for trend < 0.05). Specifically, individuals in the highest quartile (Q4) of OBS had a 61% [OR (95% CI) = 0.39 (0.2, 0.79), p = 0.01] lower risk of infertility compared to those in the lowest quartile (Q1, serving as the reference) in Model 3. Likewise, those in Q3 of lifestyle OBS had a 58% [OR (95% CI) = 0.42 (0.19, 0.92), p = 0.032] reduced risk of infertility compared to Q1. **Supplementary Figures S1**-**3** depicted the correlation between OBS quartiles and their subgroups and female infertility under the three models as illustrated in forest plots.

**Table 3:** Association between oxidative balance score and female infertility.

Characteristic	Model 1 [(OR) (95% CI)]	P-value	Model 2 [(OR) (95% CI)]	P-value	Model 3 [(OR) (95% CI)]	P-value
**OBS (continuous)**	0.98(0.95,1)	0.062	0.97(0.94,0.99)	**0.045**	0.97(0.95,0.99)	**0.047**
**OBS Quartile**		**0.002**		**0.009**		**0.01**
Q1	ref	—	ref	—	ref	—
Q2	0.59(0.36,0.96)	**0.033**	0.6(0.36,0.99)	**0.047**	0.65(0.41,1.03)	0.069
Q3	0.95(0.53,1.71)	0.9	0.89(0.51,1.56)	0.7	0.96(0.56,1.62)	0.9
Q4	0.41(0.22,0.75)	**0.005**	0.38(0.19,0.77)	**0.008**	0.39(0.2,0.79)	**0.01**
**p for trend**		**0.035**		**0.027**		**0.033**
**OBS Dietary**	0.98(0.95,1.01)	0.2	0.98(0.95,1.01)	0.11	0.98(0.95,1.01)	0.11
**OBS Dietary Quartile**		0.391		0.424		0.489
Q1	ref	—	ref	—	ref	—
Q2	0.71(0.38,1.3)	0.3	0.72(0.39,1.33)	0.3	0.78(0.44,1.37)	0.4
Q3	0.87(0.47,1.61)	0.6	0.79(0.44,1.4)	0.4	0.84(0.5,1.41)	0.5
Q4	0.66(0.38,1.13)	0.13	0.62(0.34,1.13)	0.12	0.65(0.36,1.16)	0.14
**p for trend**		0.2		0.14		0.2
**OBS Lifestyle**	0.82(0.7,0.97)	**0.019**	0.82(0.67,0.99)	**0.049**	0.83(0.69,1.01)	0.068
**OBS Lifestyle Quartile**		**<0.001**		**<0.001**		**0.002**
Q1	ref	—	ref	—	ref	—
Q2	0.98(0.54,1.77)	>0.9	1.06(0.55,2.04)	0.9	1.11(0.58,2.15)	0.7
Q3	0.4(0.2,0.8)	**0.01**	0.39(0.18,0.86)	**0.02**	0.42(0.19,0.92)	**0.032**
Q4	0.43(0.22,0.83)	**0.014**	0.41(0.17,0.95)	**0.037**	0.44(0.19,1)	0.051
**p for trend**		**0.003**		**0.011**		**0.016**

OR, odds ratio; 95% CI, 95% confidence interval; ref, reference.

Model 1: univariable logistic regression model; Model 2: multivariable logistic regression model adjusted for age, PIR, smoking, and drinks; Model 3: multivariable logistic regression model adjusted for age, PIR, smoking, drinks, PID, and female hormones. Values in bold indicate statistical significance.

### Nonlinear relationship exploration

**Figure 2:**
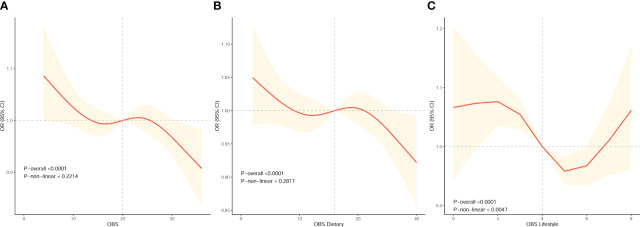
The dose-response association between OBS and the risk of infertility in female based on RCS analysis. **(A–C)** RCS analysis based on weighted multivariable logistic regression after adjusting covariates (Model 3) to investigate the nonlinear association between OBS, OBS in dietary components, and OBS in lifestyle components and female infertility prevalence.

We conducted RCS analysis using weighted multivariable logistic regression to assess the correlation between OBS and infertility risk in female while adjusting covariates. The results revealed significant associations between OBS, dietary OBS, and lifestyle OBS with infertility risk (all P-overall < 0.0001). Particularly, we observed an inverse correlation between OBS (including dietary OBS) and the prevalence of infertility, as depicted by the spline smoothing plot, which indicated that higher OBS (including dietary OBS) corresponded to a reduced prevalence of infertility (P non−linear > 0.05) (**Figures 2A, B**). However, lifestyle OBS demonstrated a nonlinear association with infertility risk (P non−linear = 0.0047). Moreover, the inflection point for this nonlinear relationship between lifestyle OBS and infertility was found to be at point 4 (**Figure 2C**). Following this, we conducted a threshold effect analysis centered on this inflection point. Notably, lifestyle OBS was significantly negatively associated with female infertility risk in an overall trend both before and after the inflection point when lifestyle OBS was in the range of 2 [OR (95% CI) = 1.076 (1.036, 1.119)] to 5 [OR (95% CI) = 0.958 (0.935, 0.982)].

### Subgroup analysis

**Figure 3:**
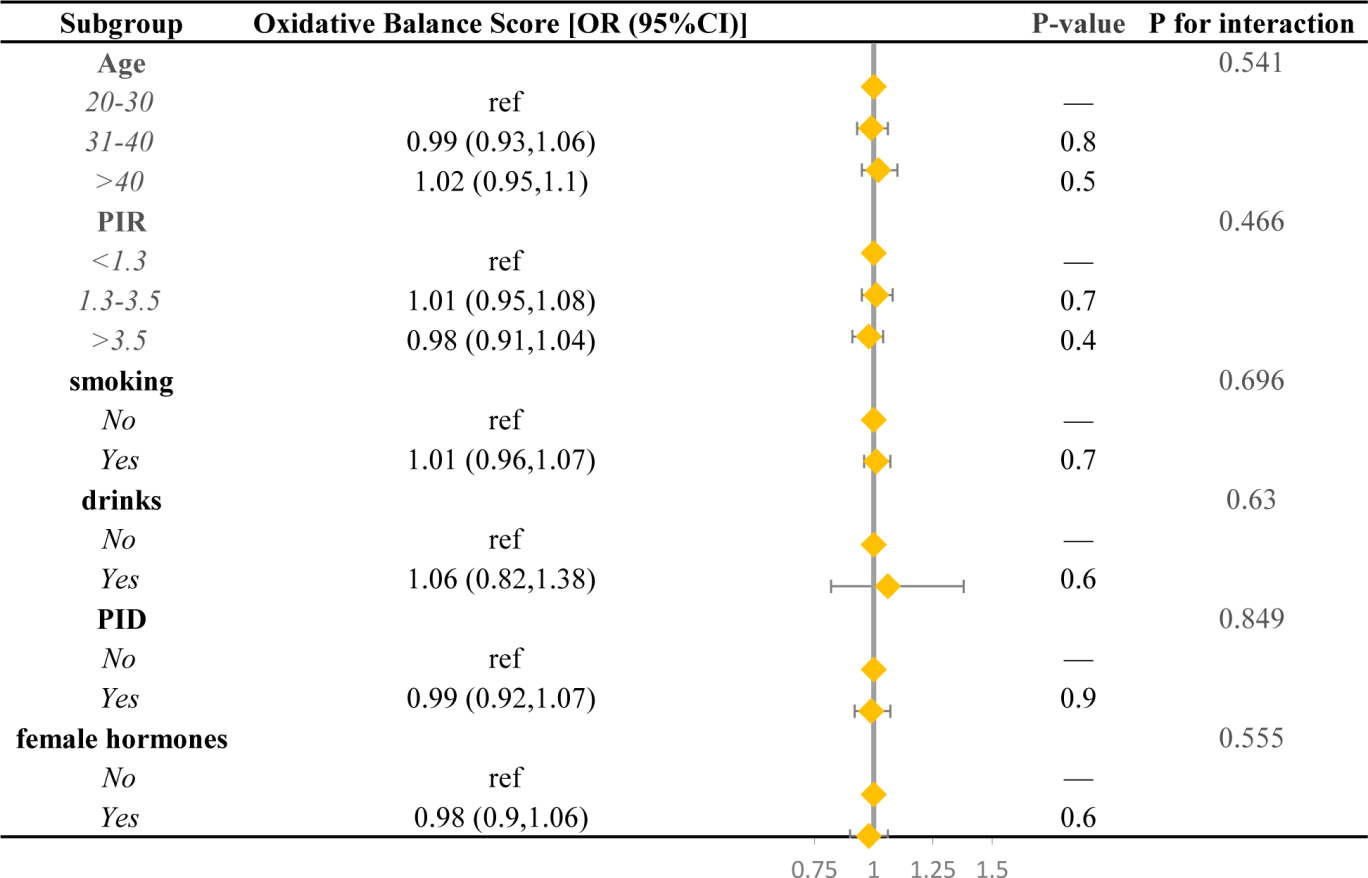
Forest plot for stratified analysis. OR, odds ratio; 95% CI, 95% confidence interval.

To explore whether the correlation between OBS and infertility risk in female remains consistent across different subgroups, we conducted a stratified analysis. The prevalence of infertility risk decreased with each unit increase in OBS in the PIR, PID, and female hormones subgroups. Conversely, a rise in OBS corresponded to an elevated risk of infertility in the age, smoking, and drinks subgroups. Notably, the statistical difference was not significant, and the P-value for interaction was greater than 0.05 across all subgroups (age, PIR, smoking, drinks, PID, and female hormones), suggesting that our findings are uniformly consistent in all subgroups, as illustrated in **Figure 3**.

### Sensitivity analysis

To ensure the reliability of our results, we carried out a sensitivity analysis. By sequentially excluding each component of the OBS, we obtained comparable outcomes, as presented in **Table 4**. These results demonstrated the robustness and stability of our findings.

**Table 4:** Sensitivity analysis to assess the effects of individual OBS components on female infertility.

Characteristic	Model 3 [(OR) (95% CI)]	P-value
**OBS Model 3**	0.97(0.95,0.99)	**0.047**
OBS excluding Dietary fiber	0.97(0.94,0.99)	**0.049**
OBS excluding Beta-carotene	0.97(0.95,1)	0.06
OBS excluding Riboflavin	0.97(0.94,0.99)	**0.046**
OBS excluding Niacin	0.97(0.94,0.99)	**0.028**
OBS excluding Vitamin B_6_	0.97(0.94,1)	0.055
OBS excluding Total folate	0.97(0.94,0.99)	**0.044**
OBS excluding Vitamin B_12_	0.97(0.94,0.99)	**0.035**
OBS excluding Vitamin C	0.97(0.95,1)	0.07
OBS excluding Vitamin E	0.97(0.94,0.99)	**0.037**
OBS excluding Calcium	0.97(0.94,0.99)	**0.04**
OBS excluding Magnesium	0.97(0.94,0.99)	**0.038**
OBS excluding Zinc	0.97(0.94,1)	0.056
OBS excluding Copper	0.97(0.94,0.99)	**0.041**
OBS excluding Selenium	0.97(0.94,0.99)	**0.043**
OBS excluding Total fat	0.98(0.95,1)	0.059
OBS excluding Iron	0.97(0.94,0.99)	**0.046**
OBS excluding Cotinine	0.97(0.95,1)	0.053
OBS excluding Alcohol	0.97(0.95,0.99)	**0.049**
OBS excluding Body mass index	0.98(0.95,1)	0.1
OBS excluding Physical activity	0.97(0.95,0.99)	**0.043**

Model 3: multivariable logistic regression model adjusted for age, PIR, smoking, drinks, PID, and female hormones. Values in bold indicate statistical significance.

## Discussion

To elucidate the relationship between OBS and female infertility, we conducted a cross-sectional analysis involving 1410 participants from the NHANES database. We consistently observed a negative correlation between OBS and infertility risk in female, implying that increased antioxidant intake and decreased exposure to pro-oxidant, reflected by higher OBS levels, could potentially mitigate the chance of developing infertility. Even after considering potential confounding factors, this correlation remained significant, and similar effects existed for both dietary OBS and lifestyle OBS, reinforcing the critical influence of OBS on the initiation and advancement of female infertility. Therefore, the higher the OBS, the more favorable the reproductive outcomes. Our findings underscored the significance of adopting an antioxidant-rich diet and healthy lifestyle, particularly for reproductive health.

This study represented the first attempt to examine the connection between OBS and female infertility, and it highlighted the inverse association between OBS levels resulting from dietary intake and lifestyle and the risk of infertility. Infertility is a complex condition influenced by multiple factors, including lifestyle, dietary habits, and nutrition (39). An imbalance between the body’s antioxidant protection and the release of ROS leads to OS production, which can impact fertility (40). Various studies have pointed out the heightened levels of OS in individuals with infertility. Polak et al. discovered significantly elevated peritoneal fluid lipid peroxide levels in women with unexplained infertility in comparison to the control group (41). Similarly, Wang et al. also confirmed noteworthy differences in ROS levels between patients with unexplained infertility and controls in processed peritoneal fluid (42). Additionally, Borowiecka et al. revealed that increased lipid and protein peroxidation levels in follicular fluids might adversely affect *in vitro* fertilization outcomes (43). In conclusion, the excessive production of ROS triggers OS events that can have a significant impact on the female reproductive process. Our research results align with the present knowledge regarding the role of OS in infertility pathogenesis, as the protective effect of higher OBS against the development of infertility was revealed.

Numerous dietary components have been demonstrated to be linked to reproductive function. For instance, a cross-sectional study of Australian women aged 18-44 revealed that insufficient levels of vitamin B_12_ have an adverse impact on women’s reproductive health (44). Another study indicated that inadequate dietary vitamin D is associated with decreased fertility in female rodents, which is rectified following vitamin D supplementation (45). Vitamin E assists in safeguarding the ovarian surface epithelium from oxidative damage, while magnesium aids in the binding of follicle-stimulating hormone to ovarian receptors (46). According to the Nurses’ Health Study-II cohort, the intake of multivitamins is inversely associated with anovulatory dysfunction in women (47). Folate is crucial in human reproduction as it affects DNA, amino acid, and methionine synthesis (48). Iron is traditionally viewed as a pro-oxidant (49), however, recent studies revealed that iron could also exhibit antioxidant properties under certain conditions (50, 51). Investigation examining the impact of minerals on ovulatory infertility demonstrated positive outcomes following iron supplementation (52). The sensitivity analysis suggested there was still a significant negative association between OBS and female infertility when iron was excluded, although this could improve some odds for the OBS lifestyle or decrease the level of statistical significance. Zinc plays a critical role in regulating various physiological processes of female germ cell growth, fertility, and pregnancy (53). A case–control study conducted by Maeda et al. revealed a significant correlation between infertility and low selenium levels, implying protective properties (54). A research by Rashidi et al. demonstrated that female mice lacking 25-hydroxyvitamin D 1α-hydroxylase [1α(OH)ase(-/-)] experienced impaired reproductive function when their blood calcium and phosphorus levels were low. This manifested as abnormalities in follicle maturation, corpus luteum formation, and underdeveloped uterine tissues, ultimately leading to infertility. Remarkably, supplementing their diet to restore normal serum calcium and phosphorus concentrations successfully mitigated these reproductive deficiencies, allowing the female 1α(OH)ase(-/-) mice to regain fertility (55). Furthermore, a study by Rashidi et al. proved improved follicular response and menstrual disturbances in infertile PCOS patients who received calcium and vitamin D treatments (56). Tiboni et al. illustrated that smoking can lead to decreased levels of carotene in the follicular microenvironment, which in terms of reproductive outcome is reflected in a significant decrease in fertilization rate in smokers. This suggested carotene plays a pivotal role in protecting the follicular microenvironment from oxidative stress. Depletion of carotene in smokers’ follicular fluid may be a contributing factor to their reduced reproductive potential (19). Research has indicated that increased consumption of soluble fiber is correlated with a higher likelihood of conception. The positive effects of a high-fiber diet on female reproduction may be attributed to its blood sugar-lowering properties (57). Additionally, concerning lifestyle factors, a meta-analysis of 12 studies has documented a notably increased OR for infertility among smokers, accompanied by a prolonged time to conception, possibly due to the activation of OS mechanisms (58, 59). A study from Denmark revealed an elevated infertility risk in women aged 30 and above who consume seven or more alcoholic beverages weekly, suggesting that alcohol may exacerbate age-related infertility (16). Obese women generally experience prolonged time to conceive and face a heightened risk of miscarriage compared to their leaner peers (18). A meta-analysis has provided evidence for an inverse correlation between PA and infertility risk. Moderate to high levels of PA were found to significantly mitigate the overall risk of infertility, establishing PA as a widely recognized protective factor (20). It is evident that a variety of dietary and lifestyle factors have differing impacts on reproductive function. How can we merge these factors to analyze their influence on reproduction? OBS can provide some insights into this question. OBS evaluates individuals’ overall balance of oxidation-reduction status, which represents the overall burden of OS (60, 61), and is linked to the onset and progression of pathological processes that impact female reproductive health (62). While normal levels of ROS are vital for regulating various physiological functions, including folliculogenesis, oocyte maturation, and fetoplacental development (63), excessive ROS can be damaging and closely associated with reproductive outcomes. Therefore, tight control over ROS generation is a pivotal process. The role of OS in the development of female infertility has captivated researchers for many years and is visible in various aspects of ovary and uterus function. ROS impact several ovarian physiological processes, such as steroidogenesis, oocyte maturation, blastocyst formation, implantation, luteolysis, and luteal maintenance during pregnancy, among others. Additionally, ROS serves as a key modulator of ovarian germ cell and stromal cell physiology (62, 64). OS has been implicated in PCOS, the prevalent endocrine disorder among women of reproductive age, characterized by ovulatory dysfunction, hyperandrogenism, and polycystic ovaries (65). A study conducted by Hilali et al. revealed that individuals with PCOS exhibit elevated serum prolidase activity, along with increased total oxidant status and OS indices (66). Furthermore, around 10 percent of women of childbearing age suffer from endometriosis, a chronic condition causing pelvic pain and infertility due to the growth of endometrial tissue outside the uterine cavity (67). A report suggested that endometriotic cells exhibit enhanced endogenous ROS production and alterations in ROS detoxification pathways and that antioxidant molecules could be used as an effective and adjunct therapy in the comprehensive management of endometriosis (68). Our study demonstrated that OBS, both from the dietary and lifestyle components, is linked to a reduced prevalence of infertility in female. These findings further underscored the significance of OBS in evaluating antioxidant capacity in patients at risk of infertility.

Following adjustment for included confounders (Model 3), the association between OBS, considered both as a continuous and a categorical variable, and the risk of infertility in female was examined. In comparison to Q1, individuals in Q4 with OBS showed a notable 61% reduction in the risk of infertility (P for trend < 0.05). Notably, a higher OBS associated with lifestyle factors was also independently linked to a decreased risk of infertility, resulting in a roughly 58% reduction among the Q3 population when compared to the reference group. Therefore, lifestyle OBS may contribute to a more significant reduction in infertility risk compared to dietary OBS. The precise mechanisms behind these observations remain unclear, emphasizing the need for further research to elucidate them. Subsequently, we delved deeper into the nonlinear relationship between OBS and infertility risk in female. Lifestyle OBS exhibited a nonlinear negative association with infertility risk (P non−linear < 0.05), with an inflection point observed at point 4. Threshold effect analysis indicated that the correlation trend between lifestyle OBS and infertility risk was most significant at point 4, highlighting its potential significance in managing infertility through lifestyle modification. Stratified analysis was performed to determine whether OBS still maintained its pertinent effects across diverse subgroups. The results revealed no noteworthy disparities among the subgroups (all P for interaction > 0.05), indicating the uniformity of our findings across various subpopulations and suggesting that OBS might attenuate infertility risk in individuals with diverse characteristics. A sensitivity analysis was conducted to assess the stability of the outcomes. After successively excluding each OBS component, comparable significant negative correlations with infertility risk were obtained. These findings demonstrated the steadiness of our results, and the conclusion that OBS is related to a reduced infertility risk is sturdy.

Our study’s strength lies in the utilization of a sizable, nationally representative sample, enhancing the applicability of the findings. Additionally, the all-encompassing OBS evaluation, which includes dietary and lifestyle factors, yields a thorough appraisal of antioxidant and pro-oxidant exposure. Nevertheless, our study faces certain limitations. Firstly, the nature of the study constrained the ability to ascertain a causal connection between OS and female infertility. Secondly, infertility encompassed both primary and secondary infertility, which was self-reported through questionnaires, lacking specific classification in our analysis. Thirdly, we were also unable to fully account for or remove the influence of other, unidentified variables. Fourthly, given dietary and lifestyle disparities between Western and other regions, additional corroboration of the findings is necessary in non-western countries. Lastly, reliance on 24-hour dietary recall and self-reported data might raise the potential for recall bias, possibly limiting the generalizability of our study’s conclusions to all female infertility patients. Hence, forthcoming research should consider these aspects and might integrate biomarkers of OS for heightened accuracy. Furthermore, to validate and further understand the association observed, additional comprehensive research is requisite.

## Conclusion

The examination of NHANES data from 2013-2018 unveiled a noteworthy inverse correlation between OBS and female infertility. A rise in OBS correlated with a decrease in the prevalence of female infertility. Additionally, the RCS analysis highlighted a nonlinear link between lifestyle OBS and female infertility. These findings indicated that heightened antioxidant and reduced pro-oxidant exposure might diminish the risk of infertility in female. It is crucial to conduct additional studies to confirm these findings and delve into the potential mechanisms.

## Data availability statement

The datasets presented in this study can be found in online repositories. The names of the repository/repositories and accession number(s) can be found below: https://www.cdc.gov/nchs/nhanes/index.htm.

## Ethics statement

The studies involving humans were approved by National Center for Health Statistics Ethics Review Board. The studies were conducted in accordance with the local legislation and institutional requirements. The participants provided their written informed consent to participate in this study.

## Author contributions

ZS: Conceptualization, Writing – original draft. PD: Writing – original draft. WS: Writing – original draft. XL (4th author): Writing – original draft. YL: Writing – original draft. XL (6th author): Writing – original draft. KL: Writing – review & editing. YW: Supervision, Writing – review & editing, Conceptualization.
