# Supplementary material for: Association between oxidative balance score and female infertility from the national health and nutrition examination survey 2013–2018
Source: Front Endocrinol (Lausanne). 2024 Jul 30;15:1386021. doi: 10.3389/fendo.2024.1386021 (PMC11319134; doi:10.3389/fendo.2024.1386021)
Supplement: Supplementary file 1 [file DataSheet_1.docx]

***Supplementary Material***

***Supplementary Figures and Tables***

***Supplementary Figures***


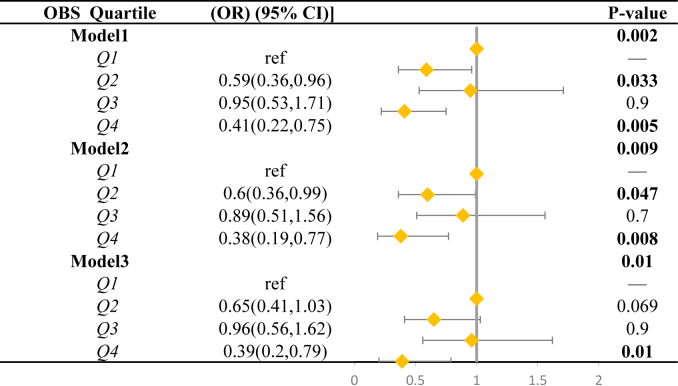


**Supplementary Figure 1**

Forest plot for OBS quartile and female infertility. OR, odds ratio; 95% CI, 95% confidence interval.

**
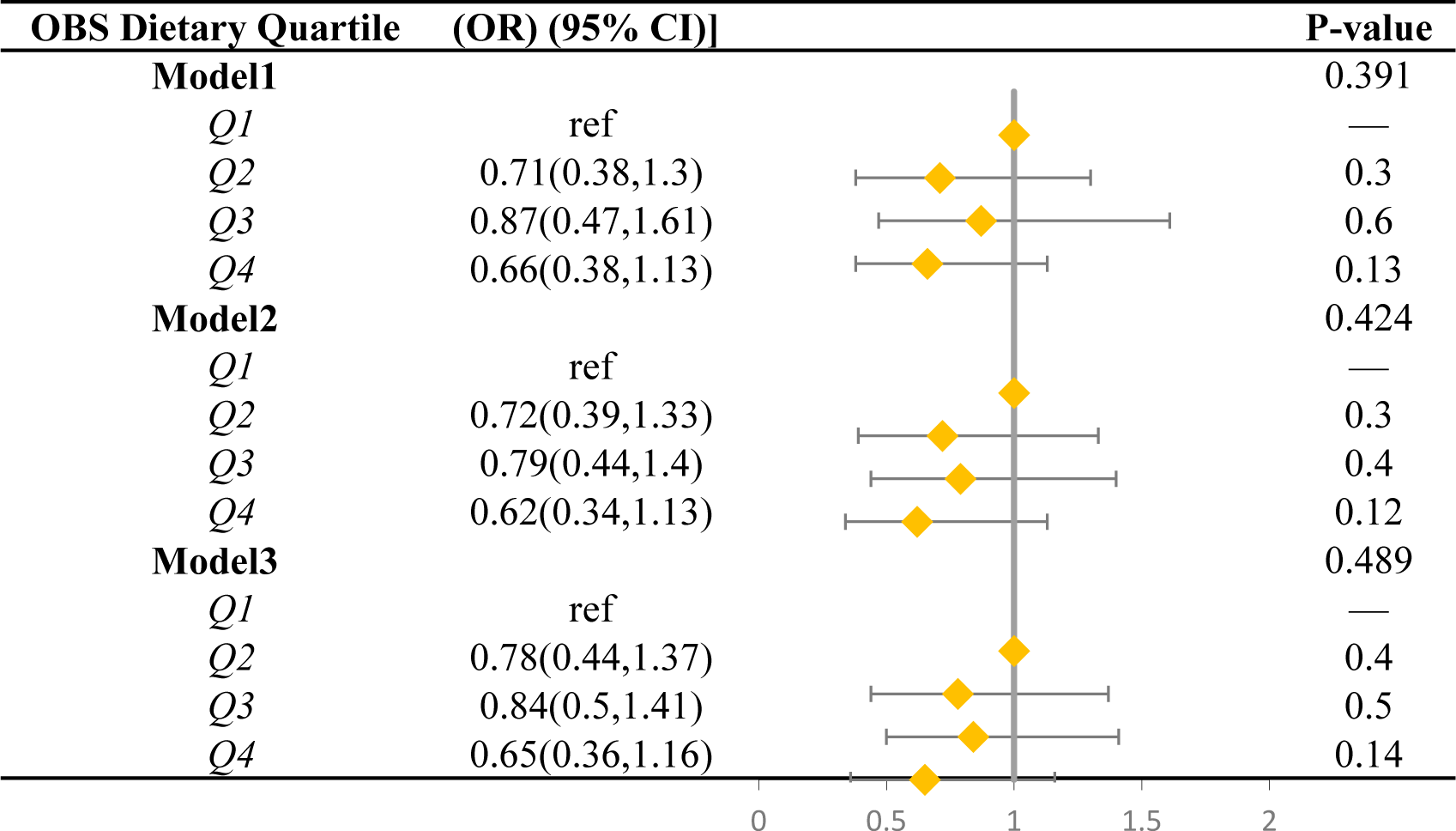
**

**Supplementary Figure 2**

Forest plot for OBS dietary quartile and female infertility. OR, odds ratio; 95% CI, 95% confidence interval.


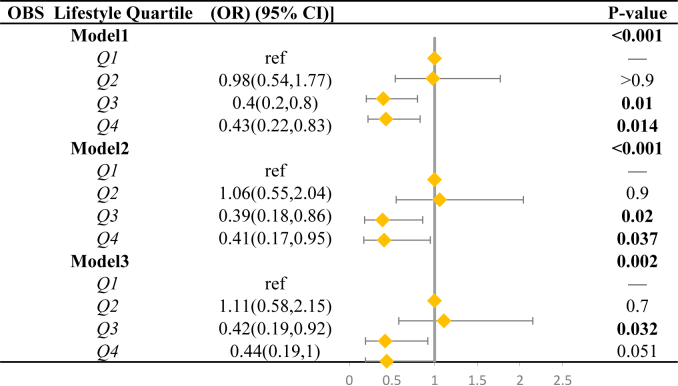


**Supplementary Figure 3**

Forest plot for OBS lifestyle quartile and female infertility. OR, odds ratio; 95% CI, 95% confidence interval.

***Supplementary Tables***

**Supplementary Table 1 Basic characteristics of participants by Oxidative Balance Score quartile.**

|  |  | **OBS Quartile** | | | |  |
| --- | --- | --- | --- | --- | --- | --- |
| **Characteristic** | **Total, N = 1410(100%)^1^** | **Q1, N = 343 (23%)^1^** | **Q2, N = 376 (26%)^1^** | **Q3, N = 350 (25%)^1^** | **Q4, N = 341 (26%)^1^** | **P-value^2^** |
| **Weighted number** | 29231894 | 6780525 | 7575463 | 7417794 | 7458112 |  |
| **Infertility** |  |  |  |  |  | **0.011** |
| *Yes* | 184 (14%) | 45 (19%) | 53 (12%) | 53 (18%) | 33 (8.5%) |  |
| *No* | 1226 (86%) | 298 (81%) | 323 (88%) | 297 (82%) | 308 (92%) |  |
| **Age (years)** | 31 (25, 37) | 30 (25, 36) | 30 (24, 36) | 31 (25, 38) | 32 (26, 38) | 0.4 |
| **Sleep hours on workdays** | 7.5 (7, 8.5) | 7.5 (6, 8.5) | 7.5 (7, 8.25) | 8 (7, 8) | 8 (7, 8.5) | 0.4 |
| **Energy (kcal)** | 1771 (1410, 2205) | 1338 (1086, 1,653) | 1643 (1380, 1943) | 1,880 (1593, 2298) | 2291 (1930, 2746) | **<0.001** |
| **BMI (kg/m^2^)** | 27 (22, 33) | 29 (24, 36) | 27 (23, 34) | 26 (22, 33) | 25 (22, 30) | **<0.001** |
| **WC (cm)** | 90 (80, 104) | 98 (85, 110) | 92 (81, 105) | 89 (78, 103) | 85 (76, 97) | **<0.001** |
| **Race/ethnicity** |  |  |  |  |  | 0.081 |
| *Mexican American* | 216 (11%) | 37 (8.4%) | 54 (11%) | 59 (10%) | 66 (14%) |  |
| *Non-Hispanic Asian* | 147 (5.2%) | 21 (3.1%) | 45 (6.8%) | 38 (4.4%) | 43 (6.2%) |  |
| *Non-Hispanic Black* | 291 (12%) | 100 (17%) | 85 (13%) | 61 (10%) | 45 (6.7%) |  |
| *Non-Hispanic White* | 553 (61%) | 136 (61%) | 141 (58%) | 140 (63%) | 136 (64%) |  |
| *Other Hispanic* | 120 (6.1%) | 23 (4.9%) | 31 (6.9%) | 30 (6.7%) | 36 (5.7%) |  |
| *Other Race* | 83 (4.8%) | 26 (6.2%) | 20 (4.1%) | 22 (5.5%) | 15 (3.7%) |  |
| **Education** |  |  |  |  |  | **<0.001** |
| *Less than 9th grade* | 32 (1.3%) | 7 (1.3%) | 8 (1.1%) | 9 (1.0%) | 8 (1.9%) |  |
| *9-11th grade* | 105 (5.2%) | 29 (7.1%) | 33 (5.9%) | 26 (4.0%) | 17 (4.1%) |  |
| *High school graduate/GED* | 247 (16%) | 87 (25%) | 64 (20%) | 59 (14%) | 37 (8.0%) |  |
| *Some college or AA degree* | 577 (39%) | 164 (47%) | 161 (40%) | 135 (36%) | 117 (35%) |  |
| *College graduate or above* | 449 (38%) | 56 (19%) | 110 (34%) | 121 (46%) | 162 (51%) |  |
| **Marital status** |  |  |  |  |  | 0.6 |
| *Living with partner* | 203 (13%) | 52 (14%) | 49 (16%) | 59 (13%) | 43 (9.6%) |  |
| *Married* | 527 (40%) | 107 (36%) | 141 (40%) | 131 (40%) | 148 (45%) |  |
| *Never married* | 514 (36%) | 139 (38%) | 141 (34%) | 118 (35%) | 116 (37%) |  |
| *Separated* | 55 (2.9%) | 18 (3.2%) | 16 (3.5%) | 13 (2.6%) | 8 (2.3%) |  |
| *Widowed* | 10 (1.1%) | 3 (2.5%) | 3 (1.5%) | 3 (0.7%) | 1 (<0.1%) |  |
| *Divorced* | 101 (6.8%) | 24 (6.6%) | 26 (5.6%) | 26 (8.3%) | 25 (6.7%) |  |
| **PIR** |  |  |  |  |  | **0.009** |
| *<1.3* | 433 (25%) | 128 (32%) | 121 (29%) | 109 (24%) | 75 (16%) |  |
| *1.3-3.5* | 529 (35%) | 136 (36%) | 139 (35%) | 131 (37%) | 123 (33%) |  |
| *>3.5* | 448 (40%) | 79 (33%) | 116 (36%) | 110 (39%) | 143 (50%) |  |
| **drinks** |  |  |  |  |  | 0.071 |
| *No* | 1398 (99%) | 341 (99%) | 373 (99%) | 344 (97%) | 340 (100%) |  |
| *Yes* | 12 (1.1%) | 2 (0.5%) | 3 (0.9%) | 6 (2.7%) | 1 (0.1%) |  |
| **smoking** |  |  |  |  |  | **<0.001** |
| *No* | 1036 (75%) | 194 (61%) | 268 (73%) | 270 (77%) | 304 (88%) |  |
| *Yes* | 374 (25%) | 149 (39%) | 108 (27%) | 80 (23%) | 37 (12%) |  |
| **trouble sleeping** |  |  |  |  |  | **0.004** |
| *No* | 1065 (75%) | 232 (66%) | 289 (76%) | 273 (75%) | 271 (81%) |  |
| *Yes* | 345 (25%) | 111 (34%) | 87 (24%) | 77 (25%) | 70 (19%) |  |
| **Total PA** |  |  |  |  |  | 0.069 |
| *No* | 193 (11%) | 53 (11%) | 58 (15%) | 44 (11%) | 38 (7.0%) |  |
| *Yes* | 1,217 (89%) | 290 (89%) | 318 (85%) | 306 (89%) | 303 (93%) |  |
| **regular periods** |  |  |  |  |  | 0.4 |
| *No* | 77 (5.2%) | 18 (3.3%) | 20 (7.1%) | 23 (5.6%) | 16 (4.7%) |  |
| *Yes* | 1,333 (95%) | 325 (97%) | 356 (93%) | 327 (94%) | 325 (95%) |  |
| **PID** |  |  |  |  |  | 0.2 |
| *No* | 1,345 (96%) | 321 (93%) | 359 (96%) | 337 (97%) | 328 (98%) |  |
| *Yes* | 65 (4.1%) | 22 (6.9%) | 17 (4.3%) | 13 (3.2%) | 13 (2.2%) |  |

^1^ median (IQR) for continuous; n (%) for categorical

^2^ Wilcoxon rank-sum test for complex survey samples; chi-squared test with Rao & Scott’s second-order correction

Q, quartile; BMI, body mass index; WC, waist circumference; PIR, ratio of family income to poverty; PA, physical activity; PID, pelvic inflammatory disease.
